# Impact of diabetes and periodontal status on life quality

**DOI:** 10.1038/s41405-021-00061-w

**Published:** 2021-02-04

**Authors:** Radhika Desai, Bhushan Khobaragade, Giles McCracken, Rebecca Wassall, John J. Taylor, Susan M. Bissett, Andrew S. Pumerantz, Philip M. Preshaw

**Affiliations:** 1 University of Bristol Dental School, University of Bristol, Bristol, UK; 2 Mid Essex Trust, Southend University NHS Hospital, Southend-on-Sea, UK; 3 School of Dental Sciences, Newcastle University, Newcastle upon Tyne, UK; 4 Department of Population Health, College of Osteopathic Medicine of the Pacific, Western University of Health Sciences, Pomona, CA USA

**Keywords:** Periodontitis, Gingivitis

## Abstract

**Objectives:**

To investigate impact of periodontal status on quality of life (QoL) in type-1 (T1D) and type-2 (T2D) diabetes patients pre- and post-periodontal treatment using the Well-being Questionnaire 12 (W-BQ12) and Audit of Diabetes-Dependent Quality of Life-19 (ADDQoL-19).

**Methods:**

W-BQ12 and ADDQoL-19 were self-completed by 56 T1D and 77 T2D patients at baseline and by those with periodontitis 3 and 6-months after therapy.

**Results:**

At baseline, T1D patients had significantly higher general W-BQ12 [Median (IQR); 24.00 (20.25–27.75)] and positive well-being scores [8.00 (6.00–9.00)] (indicating better QoL) compared to T2D patients [22.00 (15.50–26.00) and 6.00 (3.50–9.00)], respectively (*p* < 0.05). Within both groups, general W-BQ12 scores did not differ significantly between patients with periodontal health, gingivitis, or periodontitis (*p* > 0.05). Significantly higher general W-BQ12 scores were observed in T1D patients at month 3 [28.00 (22.00–29.50)] compared to baseline [22.00 (17.00–24.50)] (*p* < 0.01), suggesting an initial improvement in QoL post-treatment. ADDQoL-19 identified that diabetes had greatest impact on the domain ‘freedom to eat’, with participants placing most importance on ‘family life’. No significant changes in ADDQoL-19 scores were seen post-treatment (*p* > 0.05).

**Conclusions:**

Diabetes had impacts upon aspects of life quality in both T1D and T2D patients, though any additional impact based on periodontal status was not observed when using W-BQ12 and ADDQoL-19.

## Introduction

Periodontitis is an inflammatory disease which affects the tooth-supporting structures, and can lead to periodontal attachment loss, alveolar bone loss, tooth mobility and tooth loss with concomitant effects on oral function and quality of life (QoL).^1^ Periodontitis is associated with several systemic diseases, and in particular diabetes,^2^ with studies suggesting a 2–3-fold increased risk, especially if diabetes is poorly controlled.^3^ Diabetes is a chronic metabolic disease which affects ~422 million individuals or 8.5% of the worldwide population.^4^ Type-1 diabetes (T1D) results from autoimmune destruction of the β cells of the pancreas, leading to absolute insulin deficiency, whereas type-2 diabetes (T2D) is caused by impaired insulin secretion by the β cells, and increased insulin resistance.^5^ The bi-directional link between diabetes and periodontitis is well established; not only is periodontitis considered a complication of diabetes, but presence of periodontitis has a significant impact on diabetes control, incidence and complications.^6,7^ Furthermore, it has been identified that periodontal treatment yields beneficial effects in reductions in HbA1c levels in patients with diabetes,^8^ and can be monitored over time as part of comprehensive, multidisciplinary diabetes care.^9^

Both periodontitis^10–12^ and diabetes^13–15^ are known to cause negative impacts on QoL. Patients with periodontitis experience in particular, functional limitation, pain, psychological discomfort and disability, and physical, emotional and social impacts on QoL.^11,12,16^ Diabetes also impacts health-related QoL in terms of physical, psychological and social well-being.^17,18^ Poor self-perceived oral health has been linked to health-related QoL in patients with T2D,^19^ and for these reasons more awareness of oral health in diabetes care is recommended by both the International Diabetes Federation^20^ and the American Diabetes Association.^21^ Moreover, the relevance of periodontal health in the context of holistic and interprofessional diabetes care was recently codified by a multinational working group that aimed to standardise patient-centred outcome measures reflecting the concerns and experiences of adults with T1D and T2D.^22^

Over the years, disease-specific measures of QoL have been added to generic ones, to identify trends most relevant to QoL in patients with various health-related conditions. The Well-being Questionnaire 12 (W-BQ12) is a generic QoL instrument consisting of 12 questions, to identify psychological problems in patients with diabetes.^23^ Among the diabetes-dependent QoL measures, the Audit of Diabetes Dependent Quality of Life-19 (ADDQoL-19) instrument is a widely used diabetes-specific questionnaire consisting of 19 domains, designed to capture psychological impacts of diabetes on QoL. The ADDQoL-19 has advantages over generic questionnaires as it allows patients to indicate aspects of life which apply to them and the perceived negative or positive impact and importance of these on their QoL.^24^

Most studies to date have focused on the impact of periodontal disease on QoL in patients without diabetes and only a few have focused on the impact that diabetes and periodontal disease together have on QoL. One study found that T2D patients reported negative impacts in the domains of general health and social, physical and role functioning when compared to non-diabetic patients.^19^ Another found that poorer QoL was significantly associated with clinical periodontal parameters, and diabetes patients with periodontitis had poorer QoL compared to those with gingivitis or periodontal heathy tissues.^25^ In contrast, a third study found no significant differences in QoL between patients based on periodontal diagnosis, suggesting that T2D had no impact on oral health-related QoL.^26^

Findings related to patient-centred outcomes of periodontal disease and treatment are limited, especially when considered within the context of systemic disease, such as diabetes. Accordingly, the aim of this study was to investigate the impact of periodontal status on QoL in patients with T1D and T2D pre- and post-periodontal treatment using the W-BQ12 and ADDQoL-19 instruments, given that the utility of these instruments has not been investigated in the context of periodontal health and disease previously. Furthermore, given the considerable negative impacts that diabetes and periodontitis have on QoL, we considered it important to evaluate the impact of periodontal disease and treatment on QoL in patients with diabetes, which would further inform efforts to deliver value-based health care.^22,27^

## Materials and methods

### Study design and participants

Adult patients with T1D and T2D were recruited from primary and secondary care diabetes clinics in the north east of England (Gateshead and Newcastle upon Tyne), following a convenience sampling approach. Prior to enrolment, written informed consent was obtained from all patients. The T1D and T2D patients were matched for periodontal status. Patients were given a diagnosis of periodontal health [no probing depths (PD) ≥4 mm, no clinical attachment loss (CAL), bleeding on probing (BOP) ≤15%], gingivitis (no PD ≥4 mm, no CAL, BOP >15%) or periodontitis (≥6 sites with PD ≥5 mm on separate teeth, with CAL and radiographic alveolar bone loss). Recruited patients fulfilled the following inclusion criteria: 16–50 years old, male or female, with a minimum of 20 natural teeth. Exclusion criteria included bleeding disorders, immunosuppression, conditions needing prophylactic antibiotic therapy prior to dental management, pregnancy and any prior non-surgical periodontal treatment in the last 6 weeks. Ethical approval was awarded by the National Research Ethics Service, UK (Ref. T1D 06/Q0904/16 and T2D 06/Q0904/8).

Periodontal examination was carried out by a single examiner using a UNC PCP15 manual periodontal probe. Clinical periodontal parameters recorded included PD, BOP percent, plaque index (PI),^28^ and modified gingival index (mGI).^29^ Patients diagnosed with periodontitis received oral hygiene instructions (OHI) personalised to their clinical needs, together with standard non-surgical periodontal treatment utilising full-mouth debridement approach under local anaesthesia, routinely over two visits. Follow-up appointments for early periodontal maintenance were scheduled at 3 and 6 weeks, including reinforcement of OHI and prophylaxis. Further, appointments for definitive periodontal maintenance care were scheduled at 3 and 6-months. Patients with periodontal health and gingivitis received OHI and prophylaxis at the screening appointment only and were not followed up subsequently. The W-BQ12 and the ADDQoL-19 questionnaires were used to assess QoL. All patients manually self-completed the questionnaires at the baseline appointment (month 0) prior to any treatment provided. Patients with periodontitis additionally completed both questionnaires at the 3 and 6- months maintenance appointments.

The null hypotheses in this study included, there would be (i) no differences in QoL between T1D and T2D patients irrespective of periodontal status (ii) no differences in QoL between T1D and T2D patients based on periodontal diagnosis (health, gingivitis and periodontitis) and (iii) no impact of periodontal treatment on QoL in patients with periodontitis, as determined using the W-BQ12 and the ADDQoL-19 questionnaires.

### Assessment of QoL

The W-BQ12 consists of 12 questions scored on 3 subscales: negative well-being (NWB), energy well-being (EWB) and positive well-being (PWB). Responses to each question are on a Likert response scale and range from 0 to 3. Score 0 indicates that over the past few weeks, the item applied to the individual ‘not at all’ and score ‘3’ indicates that the item applied ‘all the time’. A higher score in each subscale indicates more of the mood described (i.e. positive, energy and negative well-being), and from the subscale scores an overall general well-being (GWB) score can be generated (higher scores indicating better QoL). The ADDQoL-19 comprises two overview items: (i) a generic domain ‘present QoL’ and (ii) a diabetes-specific domain ‘impact of diabetes on QoL’. The 19 domains are related to the impact of diabetes on particular life aspects: working life, family life, friendships and social life, close personal relationships, leisure activities, holidays, local or long-distance journeys, physical health, physical appearance, self confidence, sex life, motivation to achieve things, feelings about the future, financial situation, people’s reactions, dependence on others, living conditions, freedom to eat, and freedom to drink. The 19 questions ask responders to rate how their daily life would be if they did not have diabetes. Impact rating scores range from −3 to +1 and importance rating scores range from 0 to +3. For each domain, a weighted impact score is calculated by multiplying the impact and importance rating (range −9 to +3), with lower scores indicating poorer QoL. Lastly, across all applicable domains, a mean weighted impact score (ADDQoL-19 score) is calculated for the entire measure.^30^

### Statistical analysis

All data were analysed using SPSS 24.0 statistical software. Normality of the data was tested using the Kolmogorov–Smirnov test. Means and standard deviations were determined for parametric variables and medians and interquartile ranges were determined for non-parametric variables. The Chi-squared test was used for cross-sectional analyses of discrete variables, to compare between T1D and T2D groups. The Mann–Whitney test was used for comparisons of W-BQ12 and ADDQoL-19 scores between T1D and T2D groups based on periodontal diagnosis. The Kruskal–Wallis test with post hoc Mann–Whitney tests was used to analyse the W-BQ12 and ADDQoL-19 data within the T1D and T2D groups based on periodontal diagnosis. The Friedman test with post hoc Wilcoxon Signed Rank tests was utilised for longitudinal comparisons of the effects of periodontal management. The associations between W-BQ12 and the ADDQoL-19 scores and clinical data (age, duration of diabetes, diabetes complications, HbA1c and PD) were assessed using Pearson’s correlation coefficient (*r*), if both variables were normally distributed, or otherwise using Spearman’s correlation coefficient (rho).

## Results

Fifty-six patients with T1D and 77 patients with T2D were recruited to the study. Patients were categorised according to diabetes and periodontal status: T1D-H (type-1 diabetes, periodontal health), T1D-G (type-1 diabetes, gingivitis), T1D-P (type-1 diabetes, periodontitis), T2D-H (type-2 diabetes, periodontal health), T2D-G (type-2 diabetes, gingivitis) and T2D-P (type-2 diabetes, periodontitis). Following periodontal treatment, 9 T1D-P and 18 T2D-P patients completed the longitudinal component of the study at months 3 and 6. Demographic data at month 0 are presented in Table 1. Assessment of the data revealed a significantly higher number of males in the T2D group [52 (67.5%)] compared to the T1D group [27 (48.2%)] (*p* < 0.01), and also the T2D patients were significantly older [49.00 (45.50–54.00) years] compared to the patients with T1D [28.00 (23.25–32.75) years] (*p* < 0.001). The groups were, however, well matched for ethnicity. Regarding glycaemic control, the T1D patients had significantly higher baseline HbA1c levels [8.30 (7.60–9.45)% or 67 (60–80) mmol/mol] compared to the T2D patients [7.30 (6.40–8.65)% or 56 (46–72) mmol/mol] (*p* < 0.001). No significant differences in HbA1c values were found within diabetes groups based on periodontal diagnosis (data not shown).

**Table 1:** Demographic data according to diabetes status at month 0.

	T1D (*n* = 56)	T2D (*n* = 77)
Gender *n* (%)		
Male	27 (48.2%)	52 (67.5%)**
Female	29 (51.8%)	25 (32.5%)
Age (years)	28.00 (23.25–32.75)	49.00 (45.5–54.0)***
HbA1c		
%	8.30 (7.60–9.45)***	7.30 (6.40–8.65)
mmol/mol	67 (60–80)***	56 (46–72)
Ethnicity *n* (%)		
Caucasian	55 (98.2%)	73 (94.8%)
Black	0 (0)	1 (1.3%)
Asian	1 (1.8%)	3 (3.9%)
Smoking status *n* (%)		
Current	11 (19.7%)	6 (7.8%)
Ex	32 (57.1%)	43 (55.8%)
Never	13 (23.2%)	28 (36.4%)

Data are presented as median (IQR) and frequency (%). *P* values are for cross-sectional comparisons between T1D and T2D groups.

**p < 0.01; ***p < 0.001.

The baseline (month 0) cross-sectional data are presented for all patients, whereas the longitudinal data in the periodontitis patients are only presented for those that attended at month 0, month 3 and month 6. Periodontal data at month 0 are presented in Table 2. Based on diabetes and periodontal status, within the healthy, gingivitis and periodontitis categories, no significant differences were observed between the T1D and T2D groups for any of the periodontal parameters. Within the T1D group, patients with gingivitis and periodontitis had significantly higher mean PI, mGI, PD and BOP scores compared to patients with periodontal health; and periodontitis patients had significantly higher mean mGI, PD and BOP scores compared to patients with gingivitis (all *p* < 0.05). Within the T2D group, similarly, patients with gingivitis and periodontitis had significantly higher mean PI, mGI, PD and BOP scores compared to patients with periodontal health, and patients with periodontitis had significantly higher mean PD and BOP compared to those with gingivitis (all *p* < 0.05). Following periodontal treatment (Table 3) both T1D and T2D patients with periodontitis showed statistically significant improvements in all periodontal parameters from month 0 to month 3, and from month 0 to month 6. However, no significant changes in HbA1c levels were found following treatment in either the T1D or T2D patients with periodontitis (data not shown).

**Table 2:** Periodontal data according to diabetes and periodontal status at month 0.

	T1D (*n* = 56)			T2D (*n* = 77)		
	Periodontal health (*n* = 9)	Gingivitis (*n* = 28)	Periodontitis (*n* = 19)	Periodontal health (*n* = 13)	Gingivitis (*n* = 34)	Periodontitis (*n* = 30)
PI	0.32 ± 0.25	0.93 ± 0.41^$$$^	0.98 ± 0.54^¶¶^	0.43 ± 0.22	0.91 ± 0.30^$$$^	0.79 ± 0.37^¶¶^
mGI	0.66 ± 0.55	1.60 ± 0.41^$$$^	1.96 ± 0.51^§§, ¶¶¶^	1.11 ± 0.50	1.99 ± 0.71^$$$^	1.89 ± 0.67^¶¶¶^
PD (mm)	1.73 ± 0.20	2.16 ± 0.23^$$$^	3.02 ± 0.81^§§§, ¶¶^	1.79 ± 0.09	2.12 ± 0.20^$$$^	2.96 ± 0.49 ^§§§, ¶¶¶^
BOP (%)	9.88 ± 5.67	34.66 ± 11.55^$$$^	52.66 ± 17.37^§§§, ¶¶¶^	8.53 ± 5.17	36.50 ± 13.62^$$$^	50.26 ± 23.14^§, ¶¶¶^

Data are presented as means ± SD. *P* values are for cross-sectional comparisons between T1D and T2D groups within periodontal status categories.

^$^Gingivitis compared to Health, ^$$$^*p* < 0.001.

^¶^Periodontitis compared to Health,^ ¶¶^*p* < 0.01; ^¶¶¶^*p* < 0.001.

^§^Periodontitis compared to Gingivitis,^ §^*p* < 0.05; ^§§^*p* < 0.01; ^§§§^*p* < 0.001.

**Table 3:** Periodontal data in patients with diabetes and periodontitis at month 0, month 3 and month 6.

	T1D			T2D		
	Month 0 (*n* = 9)	Month 3 (*n* = 9)	Month 6 (*n* = 9)	Month 0 (*n* = 18)	Month 3 (*n* = 18)	Month 6 (*n* = 18)
PI	0.77 ± 0.40	0.40 ± 0.24*	0.45 ± 0.23*	0.88 ± 0.39	0.56 ± 0.35**	0.56 ± 0.35***
mGI	1.87 ± 0.36	0.82 ± 0.57**	0.95 ± 0.79*	1.93 ± 0.66	1.14 ± 0.80***	1.37 ± 0.78**
PD (mm)	2.84 ± 0.85	2.39 ± 0.90*	2.42 ± 0.59*	3.11 ± 0.44	2.72 ± 0.56***	2.63 ± 0.59***
BOP (%)	47.38 ± 14.22	25.24 ± 18.86**	27.50 ± 15.26**	59.15 ± 19.59	33.06 ± 22.50***	31.32 ± 21.63***

Data are presented as means ± SD. *P* values are for paired comparisons from month 3 and month 6 to month 0.

*p < 0.05; **p < 0.01; ***p < 0.001.

Comparing W-BQ12 scores for patients with T1D and T2D at month 0, there were no significant differences between T1D and T2D patients for NWB and EWB (*p* > 0.05) (data not shown). However, the PWB and GWB scores were significantly higher for patients with T1D [8.00 (6.00–9.00)] and 24.00 (20.25–27.75)] (indicating better QoL) than patients with T2D [6.00 (3.50–9.00) and 22.00 (15.50–26.00)] (*p* < 0.05). When W-BQ12 scores were categorised according to diabetes and periodontal status (Table 4), W-BQ12 scores did not significantly differ between the health, gingivitis, and periodontitis patients. We identified that T2D-G patients had significantly higher NWB scores (indicating poorer QoL) compared to T1D-G patients (*p* < 0.05). Table 5 shows W-BQ12 scores following periodontal treatment. Within the T1D group, significantly higher EWB, PWB and GWB scores were observed at month 3 compared to month 0 (all *p* < 0.05), suggesting an initial improvement in QoL post-treatment, although this benefit was no longer present at month 6. At baseline, analysis based on gender, revealed that only in the T1D group, males had a significantly higher GWB scores [26.00 (22.00–29.00)] compared to females [23.00 (19.00–25.00)] (*p* < 0.05).

**Table 4:** W-BQ12 scores in patients with T1D and T2D at month 0 categorised by periodontal status.

	T1D (*n* = 56)			T2D (*n* = 77)		
	Periodontal health (*n* = 9)	Gingivitis (*n* = 28)	Periodontitis (*n* = 19)	Periodontal health (*n* = 13)	Gingivitis (*n* = 34)	Periodontitis (*n* = 30)
Negative well-being (NWB)	2.00 (0.00–2.50)	1.00 (0.00–3.00)*	2.00 (0.00–5.00)	2.00 (0.50–7.50)	3.00 (0.75–5.00)*	1.00 (0.00–3.50)
Energy well-being (EWB)	6.00 (5.00–8.50)	6.00 (5.00–7.00)	6.00 (5.00–7.00)	3.00 (2.00–8.00)	5.50 (3.00–7.00)	6.00 (5.00–7.00)
Positive well-being (PWB)	8.00 (6.00–8.00)	8.00 (6.00–9.00)	8.00 (7.00–9.00)	4.00 (3.00–9.50)	7.50 (4.00–8.00)	6.50 (3.00–10.00)
General well-being (GWB)	24.00 (23.50–25.50)	24.00 (19.25–28.00)	23.00 (20.00–28.00)	17.00 (10.00–29.00)	23.00 (15.00–24.25)	23.00 (18.75–27.25)

Data are presented as medians (interquartile range). *P* values are for cross-sectional comparisons between T1D and T2D groups within periodontal status categories. *p < 0.05 when comparing scores between T1D and T2D.

**Table 5:** W-BQ12 scores in patients with periodontitis, and with T1D and T2D at month 0, month 3 and month 6.

	T1D			T2D		
	Month 0 (*n* = 9)	Month 3 (*n* = 9)	Month 6 (*n* = 9)	Month 0 (*n* = 18)	Month 3 (*n* = 18)	Month 6 (*n* = 18)
Negative well-being (NWB)	2.00 (1.00–5.00)	1.00 (0.00–3.00)	2.00 (0.00–3.00)	1.00 (0.00–3.00)	1.50 (0.00–3.25)	1.00 (0.00–3.00)
Energy well-being (EWB)	5.00 (3.00–6.00)	7.00 (4.50–8.50)*	5.00 (3.50–8.00)	6.00 (4.75–8.00)	6.00 (4.00–8.25)	7.00 (4.00–9.25)
Positive well-being (PWB)	8.00 (6.00–8.50)	8.00 (7.50–10.50)*	7.00 (5.50–8.00)	7.50 (3.00–10.00)	8.00 (5.00–9.00)	8.00 (5.00–10.00)
General well-being (GWB)	22.00 (17.00–24.50)	28.00 (22.00–29.50)**	23.00 (21.00–25.00)	23.50 (18.75–28.25)	24.50 (19.75–29.25)	25.00 (18.00–30.25)

Data are presented as medians (interquartile range). *P* values are for paired comparisons of month 3 and month 6 to month 0.

*p  < 0.05; **p  < 0.01.

Table 6 shows the ADDQoL-19 scores for patients with T1D and T2D at month 0. Of note, in both groups, ADDQoL-19 identified that diabetes had the greatest impact on the domain of ‘freedom to eat’ and the patients placed most importance on ‘family life’. Comparing both groups, diabetes had significantly greater negative impacts on the ‘working life’ and ‘freedom to drink’ domains in the T1D patients compared to the T2D patients (*p* < 0.05 and *p* < 0.01, respectively), and a significantly greater negative impact on the ‘feelings about the future’ domain in the T2D patients compared to the T1D patients (*p* < 0.001). Interestingly, this study highlighted some differences between both patient groups, with T1D patients’ diabetes having significantly greater negative impact and patients giving more importance to domains of ‘working life’, ‘holidays’, ‘friendship and social life’ and ‘freedom to drink’ and also placing more importance on ‘physical appearance’ compared to T2D patients. Weighted impact scores revealed that diabetes had a significantly greater negative impact on ‘feelings about the future’ in the T2D patients compared to T1D patients (*p* < 0.001). No significant differences were found for the ADDQoL-19 score and overview item 2 between patients with T1D (−1.81 ± 1.40 and −1.23 ± 0.87) and T2D (−1.55 ± 1.59 and −1.03 ± 1.04) (*p* > 0.05). However, significant differences in overview item 1 were found between T2D patients (0.88 ± 1.22) and T1D patients (1.45 ± 0.76) (*p* < 0.01), suggesting that although both groups did not perceive their general life quality as ‘bad’, ‘very bad’ or ‘extremely bad’, both groups felt that in general their QoL was ‘neither good nor bad’ to ‘good’ for T2D patients and ‘good’ to ‘very good’ for T1D patients. When ADDQoL-19 scores were categorised according to diabetes and periodontal status, scores did not significantly differ between patients with periodontal health, gingivitis, and periodontitis (data not shown). At baseline, analysis based on gender, revealed that females had a significantly greater negative impact on the ‘physical appearance’, ‘dependence on others’ and ‘freedom to eat’ domains (*p* < 0.05) (data not shown). Following periodontal treatment in patients with periodontitis, no significant changes in ADDQoL-19 scores were found over the course of the study in either the T1D (ADDQoL score month 0: −2.05 ± 2.03; month 3: −1.94 ± 1.91; month 6: −2.33 ± 2.21) or T2D patients (ADDQoL score month 0: −1.05 ± 1.14; month 3: −1.27 ± 1.26; month 6: −1.08 ± 1.15) (all *p* > 0.05). Finally, no significant associations were found between W-BQ12 and the ADDQoL-19 scores and clinical data (age, duration of diabetes, diabetes complications, HbA1c and mean PD) (all *p* > 0.05).

**Table 6:** ADDQoL-19 scores in patient with T1D and T2D at baseline (month 0).

ADDQoL-19 domains	T1D (*n* = 56) Impact rating	T2D (*n* = 77) Impact rating	T1D (*n* = 56) Importance rating	T2D (*n* = 77) Importance rating	T1D (*n* = 56) Weighted impact score	T2D (*n* = 77) Weighted impact score
Leisure activities	−1.16 ± 1.04	−0.87 ± 0.85	1.91 ± 0.77	1.74 ± 0.84	−2.43 ± 2.56	−1.73 ± 2.13
Working life	−0.86 ± 0.97	−0.51 ± 0.84*	2.40 ± 0.76	1.75 ± 1.19***	−2.22 ± 2.72	−1.20 ± 2.12*
Journeys	−0.96 ± 1.01	−0.70 ± 1.03	1.59 ± 1.07	1.34 ± 1.08	−2.00 ± 2.60	−1.31 ± 2.43
Holidays	−1.19 ± 0.96	−0.85 ± 0.99	2.36 ± 0.74	1.85 ± 0.93**	−2.79 ± 2.59	−1.80 ± 2.55*
Physical health	−1.02 ± 0.92	−1.01 ± 1.04	2.13 ± 0.79	1.88 ± 0.78	−2.18 ± 2.36	−2.03 ± 2.39
Family life	−0.55 ± 0.85	−0.68 ± 0.88	2.71 ± 0.59	2.79 ± 0.53	−1.46 ± 2.46	−1.97 ± 2.54
Friendship and social life	−0.73 ± 0.90	−0.55 ± 0.82	2.61 ± 0.53	2.16 ± 0.85***	−1.96 ± 2.57	−1.32 ± 2.06
Personal relationships	−0.54 ± 0.76	−0.51 ± 0.84	2.64 ± 0.72	2.50 ± 0.80	−1.39 ± 2.12	−1.35 ± 2.28
Sex life	−0.40 ± 0.68	−0.65 ± 0.98	2.38 ± 0.65	2.10 ± 0.91	−0.85 ± 1.58	−1.58 ± 2.70
Physical appearance	−0.64 ± 0.90	−0.68 ± 1.00	2.05 ± 0.88	1.58 ± 0.85**	−1.55 ± 2.49	−1.36 ± 2.15
Self confidence	−0.64 ± 0.82	−0.58 ± 0.94	2.21 ± 0.73	2.04 ± 0.79	−1.46 ± 2.21	−1.43 ± 2.58
Motivation	−0.66 ± 0.90	−0.54 ± 0.90	2.14 ± 0.72	1.88 ± 0.89	−1.52 ± 2.40	−1.16 ± 2.12
People’s satisfaction	−0.30 ± 0.63	−0.27 ± 0.62	1.75 ± 0.94	1.57 ± 0.86	−0.64 ± 1.59	−0.55 ± 1.47
Feelings about the future	2.18 ± 0.77	−1.13 ± 1.10***	2.18 ± 0.77	2.00 ± 0.89	5.32 ± 2.97	−2.66 ± 3.05***
Financial situation	−0.25 ± 0.69	−0.36 ± 0.81	2.16 ± 0.71	1.96 ± 0.82	−0.46 ± 1.33	−0.82 ± 1.93
Living conditions	−0.21 ± 0.53	−0.37 ± 0.75	2.29 ± 0.62	2.05 ± 0.78	−0.54 ± 1.40	−0.80 ± 1.79
Dependence on others	−0.58 ± 0.80	−0.35 ± 0.69	2.00 ± 0.83	1.99 ± 0.95	−1.36 ± 2.21	−0.71 ± 1.63
Freedom to eat	−1.83 ± 1.03	−1.65 ± 1.02	1.92 ± 0.81	1.71 ± 0.86	−3.77 ± 3.00	−3.30 ± 2.96
Freedom to drink	−1.72 ± 1.10	−1.23 ± 1.02**	1.64 ± 1.04	1.34 ± 0.85	−3.28 ± 3.29	−2.10 ± 2.31*

Data are presented as means ± SD. *P* values are for cross-sectional comparisons between T1D and T2D groups for impact ratings, importance ratings and weighted impact scores.

*p  < 0.05; **p  <  0.01; ***p  < 0.001.

## Discussion

This study investigated the impact of periodontal status on QoL in patients with T1D and T2D pre- and post-periodontal treatment using two validated instruments routinely used to assess the impact of diabetes on QoL, the W-BQ12 and the ADDQoL-19.

The comparison of demographic data showed a significant difference in age between the groups, with the T2D patients being significantly older (median 49 years) than the T1D patients (median 28 years). This could be relevant in this study, given the findings of some significant differences between the T1D and T2D patients, which potentially could be related to age. For example, the T1D patients were more concerned about their ‘working life’, ‘holidays’, ‘friendship and social life’, ‘freedom to drink’ and ‘physical appearance’ compared to the T2D patients, as highlighted by the ADDQoL-19 instrument (Table 6). On the other hand, the T2D patients were more anxious about their future compared to the T1D patients.

Patients were allocated to the clinical groupings of health, gingivitis, and periodontitis according to their baseline clinical data. Although no significant differences were found in clinical periodontal parameters between the T1D and T2D groups, it is noteworthy that overall, the T1D patients had significantly higher baseline HbA1c levels [8.30 (7.60–9.45)% or 67 (60–80) mmol/mol] compared to the T2D patients [7.30 (6.40–8.65)% or 56 (46–72) mmol/mol]. Clearly, this is indicative of poorer glycaemic control in this population of T1D patients compared to their T2D counterparts. This may be related to aspects such as their age, the length of time since their diabetes was diagnosed, and their ongoing efforts to achieve a good level of control, as well as the underlying pathogenic processes in both disease states. Further studies would be required to investigate this in more detail. The longitudinal analyses indicated that periodontal treatment had a significant positive effect upon the clinical periodontal parameters (as would be expected), yielding clinically relevant benefits. Although successful periodontal treatment has been clearly associated with reductions in HbA1c in previously reported randomised controlled trials,^8^ no significant changes in HbA1c were observed following the periodontal treatment in the present study, though it was not designed or powered to test the impact of periodontal therapy on glycaemic control.

Pre-treatment analyses of the W-BQ12 data revealed significantly better QoL in T1D patients compared to T2D patients, which may be possibly associated with the significant differences in age between the groups. However, in both the T1D and T2D patients, no significant differences were observed in QoL between those with periodontal health, gingivitis and periodontitis (Table 4), suggesting that either the patients’ periodontal status had no effect on their perception of QoL or that the W-BQ12 is not sensitive to changes in QoL that may arise as a result of worsening periodontal conditions. It is of interest that longitudinal analyses of the W-BQ12 data in the T1D patients with periodontitis revealed a significant improvement in QoL at 3 months post-treatment, compared to the pre-treatment scores. However, such a difference was no longer apparent at month 6, suggesting that any positive impact of periodontal treatment on QoL was of relatively short duration. In this respect, our data are similar to the results of previous research which have also showed that successful periodontal treatment has a positive impact on an individual’s QoL, as measured using a variety of instruments to assess life quality.^31–34^

Analyses of the ADDQoL-19 data revealed that all patients with diabetes experienced an overall ‘good to very good’ general QoL. However, in relation to their diabetes, patients felt that their QoL would have been ‘a little better or much better’ if they did not have diabetes, thereby capturing the negative impact of diabetes on QoL in this study group. Analyses based on periodontal diagnosis suggested that patients with periodontal health, gingivitis and periodontitis had a similar perception of the effect of their diabetes on their QoL with no clear evidence of significant differences between periodontal disease categories. An interesting finding in this study was that few patients reported there to be no impact of diabetes on their QoL, and this is consistent with previous reports.^35^ In the patients who did report an impact, however, diabetes was found to have the greatest impact on their ‘freedom to eat’ and all patients placed the most importance on ‘family life’. Our findings are consistent with those of previous studies that have also reported the greatest negative impact of diabetes on the domains of ‘freedom to eat’, ‘family life’ and ‘dependence on others’.^24,35–38^ Clearly, diabetes has a strong influence on the daily lives of those with the condition, particularly in relation to dietary restrictions in order to prevent and control diabetes-related complications,^39^ and nutritional intake needs to be tailored and regulated according to patient’s age, weight, culture, lifestyle, personal preferences, intake of medications and time of day.^40,41^ Longitudinal analysis of the ADDQoL-19 data in the patients with periodontitis revealed no significant changes as a result of treatment, suggesting that QoL (as assessed by the ADDQoL-19 instrument) remained the same pre- and post-treatment in this group of patients.

The lack of significant differences observed in QoL in T1D and T2D patients based on periodontal diagnosis might be a function of the relatively small number of patients in the periodontal subgroups (or alternatively, a lack of sensitivity of the utilised instruments to identify differences in QoL resulting from different periodontal conditions). Our findings are similar to those from a previously reported study that investigated the impact of periodontal status and treatment on oral health-related QoL in patients with and without T2D, utilising the Oral Health Impact Profile-49 (OHIP-49) tool.^26^ Despite the OHIP-49 containing oral health-related questions, those researchers also found a lack of significant differences between diabetic patients with different periodontal conditions. However, within their non-diabetic control group, patients with periodontitis and gingivitis had poorer oral health-related QoL compared to those who were periodontally healthy. The authors reported this could possibly indicate that patients with diabetes are less concerned about their oral health than they are about the other health problems they need to manage as part of their diabetes care.^26^ It has been also suggested previously that as chronic diseases can have significant negative impact on patients, it is likely that their oral health would be prioritised less, especially if the benefits of achieving oral health are perceived to be inconsequential.^42^ Potentially, healthy (non-diabetic) patients might be more concerned about the signs and symptoms of periodontitis than patients with diabetes who need to address other, more pressing health issues, and this might lead to lower expectations of oral health or better coping with the impact of periodontitis.^26^ Although the OHIP is a useful measure to determine oral health-related QoL, it contains no diabetes-specific questions and therefore might not fully assess health-related QoL in patients with diabetes, which is partly why we chose to utilise W-BQ12 and ADDQoL-19 in this research.

Based on gender, at baseline, male patients with T1D had significantly better QoL compared to females, as assessed by the W-BQ12. Females also had a significantly higher impact on their physical appearance, dependence on others and their freedom to eat as assessed by the ADDQoL-19 questionnaire. The findings of the present study are consistent with previous research that suggests QoL is better among diabetic males than diabetic females.^43,44^ Our findings are also consistent with reported gender differences in health-related QoL in the general population, which suggest that males have a better perception of QoL compared to females.^45–47^

Our study has a few limitations to consider. Although the analysis of QoL in patients with T1D and T2D did reveal some significant findings of interest, we did not recruit a non-diabetic control group, hence it was not possible to make comparisons between patients with and without diabetes. Indeed, it would not be meaningful to use the ADDQoL-19 in patients who do not have diabetes, as the items in the questionnaire relate specifically to the respondent’s diabetes. To the best of our knowledge, the W-BQ12 and the ADDQoL-19 instruments have not previously been investigated for their utility in assessing QoL in diabetic patients with differing periodontal conditions. However, these questionnaires are specifically designed to evaluate overall QoL, and therefore may not fully assess oral health-related QoL. In support of this, although the patients with periodontitis showed significant improvements in clinical periodontal parameters following periodontal therapy, both questionnaires were unable to capture to a full extent any positive impact of treatment on QoL. Methodologically, it is essential to use multidimensional assessments, including both generic and diabetes-specific measures as a guide to evaluate treatment interventions and to assess QoL.^48^ We also recognise that performing statistical testing based on data from subgroups of patients with differing periodontal conditions weakened the power of the analyses. In future studies, it would be beneficial to assess QoL in sufficiently large groups of patients with different periodontal status, and with or without diabetes, utilising generic (for diabetic and non-diabetic patients) and diabetes-specific questionnaires (for diabetic patients only) and accounting for loss to follow-up so that a robust oral health-related QoL and health-related QoL assessment can be made.

## Conclusions

In conclusion, analyses of the W-BQ12 and the ADDQoL-19 questionnaire data revealed that diabetes did have impacts on certain aspects of life quality in both T1D and T2D patients, though any additional impact based on periodontal status was not observed as measured using these instruments. Furthermore, although the patients with diabetes (both T1D and T2D) showed improvements in their clinical periodontal condition following treatment, neither the W-BQ12 nor the ADDQoL-19 questionnaire appeared to be useful in capturing any impact of this improvement on QoL. These findings suggest that although these validated QoL measures are ideal for assessing health-related QoL in patients with diabetes, they do not appear to be beneficial in assessing oral health-related QoL in patients with periodontal disease and diabetes.
